# Infectious Ulcer of the Vulva of Cattle1Read before the ninth annual meeting of the Missouri Veterinary Medical Association, St. Louis, Mo., October 3 and 4, 1900.

**Published:** 1901-02

**Authors:** C. Miller

**Affiliations:** St. Louis, Mo.


### INFECTIOUS ULCER OF THE VULVA OF CATTLE.[1]

By Dr. C. Miller,
ST. LOUIS, MO.

In presenting this short paper on the above-mentioned disease my object is not to add anything particularly new or important—although this may be to many of those present—to the domain of veterinary pathology, but rather to assist if possible in furnishing some reliable literature on a disease though, so far, uncommon, yet sufficiently important to demand our studious attention.

In contributing this article, therefore, the object will be two-fold: 1. Following out the oft-repeated adage "that a careful observer makes a skilful practitioner, but his skill dies with him unless by recording his observations he thus adds to the knowledge of his profession in general, and thereby assists by such facts in building up a strong edifice of veterinary science." 2. By giving a somewhat accurate description of the disease in question and my limited experience with it I hope to make it not only easy for other members of the profession, especially the younger members, who may chance to read this article to recognize the affection immediately, but to intelligently treat the malady with professional accuracy and certainty and not be left, as your humble colleague, to diagnose, instruct, and treat a disease which he had not even heard of or seen before, hence without any previous knowledge or assistance of any kind. I well remember the embarrassing position I was placed in, and it is with a view of preventing you from being caught in a similar circumstance that I present this paper at this particular meeting.

To the best of my knowledge—and I have made some considerable inquiries along this line—there is only one article on this disease on record, and that is a paper contributed by our worthy colleague, Dr. S. Stewart, read before the M. V. V. A. in the summer of 1898 at the meeting in Kansas City. Doubtless many of you have seen or heard more or less of this affection, especially those of you who were practising during the winter of 1898 in the central Western States. This peculiar disease, as I have learned since, was quite generally distributed over four or five States, namely, Iowa, Missouri, Kansas, and Nebraska. Other States may have been visited, but not to my knowledge; if so, however,

[1] Read before the ninth annual meeting of the Missouri Veterinary Medical Association, St. Louis, Mo., October 3 and 4, 1900.

I would be glad to hear the reports of any who may have come in contact with it. In giving a short history of this disease I do not wish it to be understood that I take issue with Dr. Stewart in the particular name applied. I have called the affection by the above name simply because in my judgment and experience it best suits the pathological conditions as I found them.

My attention was called to this outbreak in the month of February, 1898, while practising in Ottumwa, Iowa. Several outbreaks occurred in the neighborhood of Blackesburg, and several stockmen came to me for information regarding its nature and probable outcome. I was simply unable to give them any really satisfactory information on the subject, and expressed a desire to go out and see the cases with my own eyes and for my benefit.

On close examination and further inquiries into the history of these particular cases I still found myself in about as deep water as ever as to a proper scientific name for the affection. I frankly admitted my ignorance of the real nature and importance of the trouble, but promised the parties if there was any literature on the subject I would certainly look it up and be ready to give them some much-needed information on my next visit. On returning home I sought wisdom and information from my esteemed brother, who had practised in the State some thirteen years, but without much success, for after exhausting the whole curriculum of veterinary terminology we came to the conclusion it must be a new disease, and hence our duty to give it a name.

The term used in this article was our mutual and final decision as a fitting appellation of this so far unknown disease, and I will leave it to your judgment as to its correctness or otherwise. The history of the first herd in which I found the disease is as follows:

The herd consisted of twenty calves, thirteen of which were heifers ranging in age from ten to fourteen months. These calves were highly fed, with open sheds for shelter, all in fine condition; in fact, were fat, but had fallen off perceptibly during this siege. These thirteen heifer calves were all more or less affected when I saw them, some eight days after the first appearance of the ulcer. The first thing noticed by the owner one morning was a wound, slightly reddened, on the lower portion of one of the vulval labia. No attention was paid to this, however, thinking an old sow had done the mischief while the calf was peacefully asleep under the shed. Not until eight of them were visibly affected was his attention sufficiently aroused and an investigation desired. At this juncture my services were summoned.

On the morning of the 8th I arrived and made a hurried examination. We secured several of the affected calves in the stable and made a careful examination of the parts affected. The first one showing signs of the disease had one lip of the vulva almost entirely eaten off, while the other labia showed a large, ulcerous wound involving the greater portion of its surface. The vulval tissues were so nearly displaced that one could hardly distinguish the animal from a male. The remaining twelve were all more or less affected, each showing different stages of the same pathological process. In the remaining five he had not noticed being affected the ulcers were so small as to escape the attention of a casual observer. I shall not attempt to give you a lengthy or elaborate description of the distinctive pathological characteristics of this specific ulcer or endeavor to describe the nature and appearances of the particular microörganism causing it, but simply and briefly describe its macroscopical appearances as they occur to my memory. This ulcer presented some marked peculiarities, namely, its tendency to spread rapidly and in the destructive agent confining its action exclusively to the vulval tissues. The infective agent, seemingly having found the proper pabulum and feasted liberally thereon, had no appetite or relish for the rich pastures which lay in such close proximity. The ulcer in almost every case started in a mere abrasion the size of a pin-head, usually on the internal surface of the labia near the border of the inferior commissure, gradually eating its way through until it appeared as a much larger denuded surface on the outside. The central part of the ulcer was of a somewhat yellowish color, surrounded by a reddened zone with very irregular borders. On irritating the surface blood would flow quite freely. The temperature of several was taken, but no appreciable disturbance could be detected. The owner said they had not been eating quite as well as usual, which doubtless accounts for their present falling off in condition, otherwise they seemed perfectly well. The treatment of these cases could not be considered of very great importance, as I found these ulcers would readily yield to even simple remedies and were not by any means obstinate to heal. However, I will recite the form of treatment adopted in these cases and leave others to profit by its use if they choose. Using carbolic acid as an antiseptic, I took hot water and cleansed the parts, tail included, thoroughly, after which I applied a strong solution of the ordinary white lotion, using a piece of cotton in applying it to the affected parts. The tail, where it came in contact with the vulva, was covered with vaseline, also the parts of the vulva not involved by the ulcer. In those cases where the ulcer was small I touched with a pencil of silver nitrate, which had the effect of arresting the action of the microörganisms at once. In fact, all the ulcers in whatever stage responded to the form of treatment and commenced healing slowly from the first application. These applications were made for four consecutive mornings, when the healing process was so far advanced that further treatment was deemed unnecessary, and within ten days they were nearly completely healed, with no traces of the disease except in those cases where the vulval tissues were so nearly completely destroyed before any application was made.

Another farmer, some ten miles from this place, had a herd of some thirty calves in which the disease made its appearance. He became somewhat alarmed and immediately disposed of them at a considerable loss. In a few days he went out and purchased a like number in the surrounding country, placing them in the same yard and feeding them on the same rations. In about ten days these calves showed symptoms of the same disease, and in a few days he was aware that they had contracted the disease. Hearing of his neighbor's experience he came to me for advice and information. It is useless to tell you I was loaded and soon relieved him of his anxiety, prescribing the same remedies as before, and sent him on his way rejoicing. I make mention of this latter outbreak simply to establish more firmly the infectious nature of the offending agent.

